# Differential Effects of Epidural Analgesia on Modes of Delivery and Perinatal Outcomes between Nulliparous and Multiparous Women: A Retrospective Cohort Study

**DOI:** 10.1371/journal.pone.0120907

**Published:** 2015-03-25

**Authors:** Tai-Ho Hung, T’sang-T’ang Hsieh, Hung-Pin Liu

**Affiliations:** 1 Department of Obstetrics and Gynecology, Chang Gung Memorial Hospital at Taipei, Taipei, Taiwan; 2 Department of Chinese Medicine, College of Medicine, Chang Gung University, Taoyuan, Taiwan; 3 Department of Anesthesiology, Chang Gung Memorial Hospital at Taipei, Taipei, Taiwan; Indiana University School of Medicine, UNITED STATES

## Abstract

**Background:**

Epidural analgesia is considered one of the most effective methods for pain relief during labor. However, it is not clear whether similar effects of epidural analgesia on the progression of labor, modes of delivery, and perinatal outcomes exist between nulliparous and multiparous women.

**Methodology/Principal Findings:**

A retrospective cohort study was conducted to analyze all deliveries after 37 weeks of gestation, with the exclusion of pregnancies complicated by multiple gestations and fetal anomalies and deliveries without trials of labor; these criteria produced a study population of n=16,852. A multivariable logistic regression model was constructed to control for confounders. In total, 7260 of 10,175 (71.4%) nulliparous and 2987 of 6677 (44.7%) multiparous parturients were administered epidural analgesia. The independent factors for intrapartum epidural analgesia included a low prepregnancy body mass index, genetic amniocentesis, group B streptococcal colonization of the genito-rectal tract, and augmentation and induction of labor. In the nulliparous women, epidural analgesia was a significant risk factor for operative vaginal delivery (adjusted odds ratio [OR] 2.14, 95% confidence interval [CI] 1.80-2.54); however, it was a protective factor against Caesarean delivery (adjusted OR 0.62, 95% CI 0.55-0.69). Epidural analgesia remained a significant risk factor for operative vaginal delivery (adjusted OR 2.17, 95% CI 1.58-2.97) but not for Caesarean delivery (adjusted OR 1.09, 95% CI 0.77-1.55) in the multiparous women. Furthermore, the women who were administered epidural analgesia during the trials of labor had similar rates of adverse perinatal outcomes compared with the women who were not administered epidural analgesia, except that a higher rate of 1-minute Apgar scores less than 7 was noted in the nulliparous women who were administered epidural analgesia.

**Conclusions/Significance:**

Intrapartum epidural analgesia has differential effects on the modes of delivery between nulliparous and multiparous women, and it is not associated with adverse perinatal outcomes.

## Introduction

Epidural analgesia is considered one of the most effective methods for pain relief during labor, and the intrapartum use of epidural analgesia has substantially increased over the previous two decades [1]. Most previous studies have demonstrated that epidural analgesia was associated with a longer second stage of labor [2–8] and a higher rate of operative vaginal delivery compared with labor without analgesia [2–5,9–11,6,7]. However, whether epidural analgesia increases a woman’s risk for a Caesarean delivery remains unclear. Some studies have shown that women who were administered epidural analgesia were more likely to have a Caesarean delivery for dystocia and fetal distress [9,10,7], whereas other studies have reported that epidural analgesia did not affect the Caesarean delivery rate [3–6]. Some studies have even indicated that epidural analgesia was a protective factor against a Caesarean delivery [2,12,11]. The reasons for these variations have been difficult to interpret and could be related to differences in the methodologies, study populations, and management styles of different hospitals.

Furthermore, it has been reported that parturients with certain demographic or pregnancy characteristics use epidural analgesia during labor more often [13,2,14]. These factors include advanced maternal age, nulliparity, a high prepregnancy body mass index (BMI), a large birth weight infant, oligohydramnios, premature rupture of membranes (PROM), and induction of labor. It is thus arguable that the increased rate of Caesarean delivery ascribed to epidural analgesia might actually be due to these maternal characteristics or to pregnancy complications, because the associations between Caesarean delivery and these risk factors have been clearly demonstrated.

Moreover, most previous studies have been performed in nulliparous women [2,12,10] or a mixture of nulliparous and multiparous women but with small sample sizes [3,9,7]. Because nulliparous women have a different baseline risk for Caesarean delivery compared with multiparous women [15], it is not clear whether similar effects of epidural analgesia on the progression of labor, modes of delivery, and perinatal outcomes exist between these two groups of women.

Therefore, the aims of this study were to study maternal demographic and pregnancy characteristics associated with intrapartum use of epidural analgesia and to investigate the effects of epidural analgesia on the modes of delivery and perinatal outcomes in a large cohort of both nulliparous and multiparous women.

## Materials and Methods

A retrospective cohort study was conducted in women who gave birth at term at Chang Gung Memorial Hospital, Taipei, Taiwan, between January 1, 2001, and December 31, 2013. Most Taiwanese are descendants of early settlers from the southeast coast of China and are genetically related to other south Asian populations. The study data were obtained from a computerized obstetrics database, which included demographic characteristics, medical and obstetric histories, and information regarding the course of the index pregnancy and perinatal outcomes [16]. The data in this database were collected by trained personnel through daily abstraction from the medical and delivery records and via postpartum interviews, if necessary, to collect supplemental information. Audits of these data were routinely performed at weekly departmental meetings. The study was approved by the Institutional Review Board of Chang Gung Memorial Hospital (No. 103–3316B); the review board also determined that informed consent was not required because of the retrospective design and the deidentification of the patients.

We analyzed all deliveries after 37 0/7 weeks of gestation (n = 23,018), excluding pregnancies complicated by multiple gestations (n = 435), fetal chromosomal or structural anomalies (n = 149), and fetal demise (n = 9). Women with a Caesarean delivery without a trial of labor for the following indications were also excluded: fetal malpresentation (n = 1434), placenta previa (n = 280), cephalopelvic disproportion including macrosomia (n = 337), a history of previous Caesarean delivery (n = 2462), previous hysterotomy for uterine fibroids (n = 183), active genital herpes or condylomatous infection (n = 14), a medical condition that would require Caesarean delivery or preclude epidural placement (n = 50), and maternal request (n = 813). Overall, 16,852 deliveries were included in the present analysis, which included 10,175 nulliparous women and 6677 multiparous women.

In our hospital, epidural analgesia is offered to all parturients during their trials of labor if there is no contraindication. The procedure is available upon request, regardless of cervical dilatation. Epidural analgesia was performed as previously described [17]. Briefly, after intravenous infusion with 500 to 1000 ml of lactated Ringer’s solution, the parturient was placed in the lateral position, and the lower lumbar epidural space was identified using the loss-of-resistance technique with an 18-gauge Tuohy needle; an epidural catheter was then inserted into the epidural space. A standardized protocol was followed during the administration of analgesia, consisting of a 10-ml initial loading dose of ropivacaine (1 mg/ml) in combination with fentanyl (7.5 μg/ml) and a continuous maintenance dose of ropivacaine (0.8 mg/ml) combined with fentanyl (2 μg/ml) at a rate of 10 ml/h after the various standard recordings (electrocardiography, automated noninvasive blood pressure, and fetal heart rate monitoring) were normal for 20 minutes. An additional 10 ml of the drug was administered at the parturient’s request as a top-up bolus. Epidural analgesia was continued through the second stage of labor. Decisions concerning obstetrical management were made by the attending obstetricians.

The following maternal demographic and pregnancy characteristics were studied: age at delivery (categorized as <20, 20–34, and >34 years old), prepregnancy BMI (categorized as <19.8, 19.8–24.2, and >24.2 kg/m^2^), conception method (natural or assisted by reproductive technology), a history of genetic amniocentesis, cigarette smoking, overt or gestational diabetes mellitus, chronic hypertension, preeclampsia, hypothyroidism, hyperthyroidism, PROM, group B streptococcus (GBS) colonization of the genito-rectal tract, fetal sex, oligohydramnios (an amniotic fluid index [AFI] <5 cm), polyhydramnios (AFI >24 cm), low birth weight (birth weight <2500 g), macrosomia (birth weight >4000 g), small-for-gestational age infants (a birth weight <10th percentile from the mean weight corrected for gestational age), and large-for-gestational age infants (a birth weight >90th percentile from the mean weight corrected for gestational age).

Our primary outcome was mode of delivery, classified as Caesarean section, operative and spontaneous vaginal delivery. Spontaneous vaginal delivery was defined as a birth without operative or instrumental assistance. Accordingly, deliveries with augmentation, episiotomy, or both were still classified as spontaneous delivery. We also examined the associations between epidural analgesia and the following adverse perinatal outcomes: neonatal death (within 28 days of birth), admission to the neonatal intensive care unit (NICU), 1-minute and 5-minute Apgar scores <7, placental abruption, acute chorioamnionitis, severe perineal injury (third and fourth degree perineal injuries), and postpartum hemorrhage (>500 ml for vaginal delivery and >1000 ml for Caesarean delivery).

Statistical analyses were performed using SPSS software, version 20.0 (SPSS Inc., Armonk, NY, USA). The continuous variables were calculated as the mean ± standard deviation. The categorical variables were calculated as the number and rate (%). Continuous parameters were compared between the groups using Student’s *t*-test, and categorical variables were compared using the χ^2^ test. A *P* value of <0.05 was considered to be statistically significant. A multivariable logistic regression model with backward elimination was constructed to control for confounders, to identify the independent factors associated with the use of epidural analgesia and to study the associations between epidural analgesia and different modes of delivery and adverse perinatal outcomes.

## Results

In our study population, 7260 of 10,175 (71.4%) nulliparous women and 2987 of 6677 (44.7%) multiparous women requested epidural analgesia during their trials of labor. Table 1 shows the maternal demographic and pregnancy characteristics of the nulliparous and multiparous women with or without the use of epidural analgesia during labor, respectively. For both the nulliparous and multiparous women, higher rates of a maternal age older than 34 years of age, a prepregnancy BMI less than 19.8 kg/m^2^, a history of genetic amniocentesis, and GBS colonization of the genito-rectal tract were identified in the women who were administered epidural analgesia compared with the women who did not receive epidural analgesia. Additionally, in the multiparous women, higher rates of a prepregnancy BMI more than 24.2 kg/m^2^ and gestational diabetes mellitus were noted in the women who were administered epidural analgesia compared with the women who were not administered epidural analgesia.

**Table 1:** Maternal demographic and pregnancy characteristics of the nulliparous and multiparous women with or without the use of epidural analgesia during labor.

Characteristics	Nulliparous women (n = 10,175)			Multiparous women (n = 6677)		
	Epidural (n = 7260)	No epidural (n = 2915)	*P*	Epidural (n = 2987)	No epidural (n = 3690)	*P*
Age (y)						
<20	36 (0.5%)	32 (1.1%)	0.001	2 (0.1%)	8 (0.2%)	0.052
20–34	6208 (85.5%)	2542 (87.1%)	0.034	2021 (67.6%)	2645 (71.7%)	<0.001
>34	1016 (14.0%)	341 (11.7%)	0.002	964 (32.3%)	1037 (28.1%)	<0.001
Prepregnancy body mass index (kg/m^2^)						
<19.8	2889 (39.8%)	1081 (37.1%)	0.014	972 (32.6%)	1044 (28.3%)	<0.001
19.8–24.2	3725 (51.3%)	1661 (54.8%)	0.001	1594 (53.3%)	2237 (60.6%)	<0.001
>24.2	646 (8.9%)	236 (8.1%)	0.172	421 (14.1%)	409 (11.1%)	<0.001
Conception assisted by reproductive technology	94 (1.3%)	29 (1.0%)	0.268	19 (0.6%)	15 (0.4%)	0.227
Genetic amniocentesis	1691 (23.3%)	551 (19.1%)	<0.001	1189 (39.8%)	1133 (30.7%)	<0.001
Cigarette smoking	17 (0.2%)	9 (0.3%)	0.517	4 (0.1%)	2 (0.1%)	0.418
Overt diabetes mellitus	4 (0.1%)	8 (0.3%)	0.007	5 (0.2%)	6 (0.2%)	0.962
Gestational diabetes mellitus	305 (4.2%)	111 (3.8%)	0.468	149 (5.0%)	133 (3.6%)	0.007
Chronic hypertension	16 (0.2%)	11 (0.4%)	0.199	4 (0.1%)	4 (0.1%)	0.765
Preeclampsia	123 (1.7%)	56 (1.9%)	0.482	24 (0.8%)	37 (1.0%)	0.471
Hypothyroidism	10 (0.1%)	5 (0.2%)	0.775	4 (0.1%)	3 (0.1%)	0.708
Hyperthyroidism	29 (0.4%)	7 (0.2%)	0.270	7 (0.2%)	4 (0.1%)	0.236
Group B streptococcal colonization	1031 (14.2%)	347 (11.9%)	0.002	559 (18.7%)	553 (15.0%)	<0.001
Premature rupture of membranes	102 (1.4%)	36 (1.2%)	0.697	23 (0.8%)	19 (0.5%)	0.214
Oligohydramnios	40 (0.6%)	26 (0.9%)	0.054	2 (0.1%)	7 (0.2%)	0.201
Polyhydramnios	10 (0.1%)	5 (0.2%)	0.775	1 (0.0%)	3 (0.1%)	0.633
Birth weight <2500 g	160 (2.2%)	111 (3.8%)	<0.001	50 (1.7%)	72 (2.0%)	0.410
Birth weight >4000 g	123 (1.7%)	47 (1.6%)	0.794	73 (2.4%)	99 (2.7%)	0.584
Small-for-gestational age	820 (11.3%)	417 (14.3%)	<0.001	206 (6.9%)	303 (8.2%)	0.040
Large-for-gestational age	385 (5.3%)	152 (5.2%)	0.803	275 (9.2%)	313 (8.5%)	0.316
Male fetus	3704 (51.0%)	1441 (49.4%)	0.152	1612 (54.0%)	1973 (53.5%)	0.692

Data are presented as the number (%).

*P* values based on χ^2^-test.

Table 2 shows the delivery characteristics of the study populations. For both the nulliparous and multiparous cohorts, the women who were administered epidural analgesia delivered at later gestational weeks and had larger birth weights and longer durations of the first and second stages of labor in the women with a vaginal delivery compared with the women who were not administered epidural analgesia. The rates of induction of labor and operative vaginal delivery were also significantly increased in the women who were administered epidural analgesia compared with the women who were not administered epidural analgesia.

**Table 2:** Delivery characteristics of the nulliparous and multiparous women with or without the use of epidural analgesia during labor.

Characteristics	Nulliparous women (n = 10,175)			Multiparous women (n = 6677)		
	Epidural (n = 7260)	No epidural (n = 2915)	*P*	Epidural (n = 2987)	No epidural (n = 3690)	*P*
Gestational age (wk)	39.1±1.0	39.0±1.1	<0.001	38.9±1.0	38.8±1.0	0.036
Birth weight (g)	3206.4±365.4	3158.8±394.5	<0.001	3272±157.1	3242±373.2	0.001
Induction of labor	2461 (33.9%)	670 (23.0%)	<0.001	705 (23.6%)	435 (11.8%)	<0.001
Augmentation of labor	3703 (51.0%)	1051 (51.5%)	0.673	1637 (54.8%)	2066 (56.0%)	0.345
Vaginal delivery, spontaneous	4970 (68.5%)	2031 (69.7%)	0.240	2765 (92.6%)	3518 (95.3%)	<0.001
Vaginal delivery, operative	896 (12.3%)	173 (5.9%)	<0.001	120 (4.0%)	64 (1.7%)	<0.001
First stage of labor (min) ^a^	271.5±210.9	153.1±156.5	<0.001	147.8±157.1	91.8±115.9	<0.001
Second stage of labor (min) ^a^	92.6±65.1	50.6±42.1	<0.001	41.8±44.0	16.2±17.2	<0.001
Caesarean delivery	1394 (19.2%)	711 (24.4%)	<0.001	102 (3.4%)	108 (2.9%)	0.287
Dysfunctional labor ^b^	1126 (15.5%)	479 (16.4%)	0.261	60 (2.0%)	42 (1.1%)	0.005
Non-reassuring FHR ^b^	383 (5.3%)	227 (7.8%)	<0.001	45 (1.5%)	57 (1.5%)	0.979
Severe preeclampsia ^b^	28 (0.4%)	21 (0.7%)	0.041	3 (0.1%)	10 (0.3%)	0.196

Data are presented as the mean±standard deviation or number (%).

FHR, fetal heart rate.

*P* values based on Student’s *t*-test or χ^2^-test.

^a^ Labor durations for the women with spontaneous or operative vaginal deliveries.

^b^ Indications for Caesarean delivery; some women had more than one indication for Caesarean delivery.

In contrast, a differential effect of epidural analgesia on the rate of Caesarean delivery was identified between the nulliparous and multiparous women. In the nulliparous cohort, a lower rate of Caesarean delivery associated with lower frequencies of non-reassuring fetal heart rate tracing (*P*<0.001) and severe preeclampsia (*P*<0.001) as the indications for the operation was noted in the women who were administered epidural analgesia compared with the women who were not administered epidural analgesia (19.2% vs. 24.4%, respectively, *P*<0.001). The rate of Caesarean delivery was not significantly different between the women who were or were not administered epidural analgesia in the multiparous cohort; however, the frequency of dysfunctional labor as an indication for Caesarean delivery was increased in the women who were administered epidural analgesia compared with the women who were not administered epidural analgesia (2.0% vs. 1.1%, *P* = 0.005).

Table 3 summarizes the rates of adverse maternal and neonatal outcomes in the study populations. In the nulliparous cohort, the newborns of the women who were administered epidural analgesia had an increased incidence of 1-minute Apgar scores less than 7 compared with the women who were not administered epidural analgesia (1.3% vs. 0.7%, respectively, *P* = 0.009), despite no differences in the rates of a 5-minute Apgar score less than 7 or other adverse maternal or neonatal outcomes between these two groups of women. In the multiparous cohort, the women who were or were not administered epidural analgesia had essentially the same rates of adverse perinatal outcomes.

**Table 3:** Adverse maternal and neonatal outcomes of the nulliparous and multiparous women with or without the use of epidural analgesia during labor.

Variables	Nulliparous women (n = 10,175)			Multiparous women (n = 6677)		
	Epidural (n = 7260)	No epidural (n = 2915)	*P*	Epidural (n = 2987)	No epidural (n = 3690)	*P*
Placental abruption	67 (0.9%)	21 (0.7%)	0.345	33 (1.1%)	36 (1.0%)	0.715
Acute chorioamnionitis	90 (1.2%)	33 (1.1%)	0.727	1 (0.0%)	1 (0.0%)	0.881
Severe perineal injury	632 (8.7%)	227 (7.9%)	0.205	57 (1.9%)	57 (1.6%)	0.256
Postpartum hemorrhage	77 (1.1%)	29 (1.0%)	0.824	51 (1.7%)	53 (1.4%)	0.430
1-minute Apgar score <7	96 (1.3%)	20 (0.7%)	0.009	13 (0.4%)	17 (0.5%)	0.877
5-minute Apgar score <7	5 (0.1%)	1 (0.0%)	0.681	1 (0.0%)	2 (0.1%)	0.691
NICU admission	112 (1.5%)	53 (1.8%)	0.364	22 (0.7%)	29 (0.8%)	0.888
Neonatal death	1 (0.0%)	0	0.526	0	3 (0.1%)	0.258

Data are presented as the number (%).

NICU, neonatal intensive care unit.

*P* values based on χ^2^-test.

To identify the factors that were associated with intrapartum use of epidural analgesia, a multivariable logistic regression analysis with adjustment for the confounding effects of various maternal and pregnancy characteristics, including maternal age, prepregnancy BMI, conception method, genetic amniocentesis, cigarette smoking, overt or gestational diabetes mellitus, chronic hypertension, preeclampsia, hypothyroidism, hyperthyroidism, GBS colonization, oligohydramnios, polyhydramnios, birth weight, small-for-gestational age, large-for-gestational age, fetal sex, induction and augmentation of labor, was performed (Table 4). For the nulliparous women, a low prepregnancy BMI (adjusted odds ratio [OR] 1.19, 95% confidence interval [CI] 1.09–1.31), a history of genetic amniocentesis (adjusted OR 1.24, 95 CI 1.09–1.41), GBS colonization (adjusted OR 1.23, 95% CI 1.07–1.40), augmentation (adjusted OR 1.66, 95% CI 1.49–1.86), and induction of labor (adjusted OR 2.49, 95% CI 2.19–2.83) were significant independent factors for the use of intrapartum epidural analgesia. For the multiparous women, the significant independent factors associated with epidural analgesia included either a low (adjusted OR 1.34, 95% CI 1.20–1.50) or high prepregnancy BMI (adjusted OR 1.36, 95% CI 1.16–1.58), a history of genetic amniocentesis (adjusted OR 1.51, 95% CI 1.33–1.72), gestational diabetes mellitus (adjusted OR 1.31, 95% CI 1.01–1.69), GBS colonization (adjusted OR 1.28, 95% CI 1.12–1.46), augmentation (adjusted OR 1.46, 95% CI 1.30–1.64), and induction of labor (adjusted OR 2.98, 95% CI 2.55–3.49).

**Table 4:** Factors associated with intrapartum use of epidural analgesia.

Variables	Nulliparous women: epidural vs. no epidural analgesia				Multiparous women: epidural vs. no epidural analgesia			
	Unadjusted OR (95% CI)	*P*	Adjusted OR^a^ (95% CI)	*P*	Unadjusted OR (95% CI)	*P*	Adjusted OR^a^ (95% CI)	*P*
Age <20 y	0.42 (0.26–0.69)	0.001	0.45 (0.27–0.73)	0.001	0.15 (0.02–1.23)	0.078	0.19 (0.02–1.51)	0.115
Age >34 y	1.23 (1.08–1.40)	0.002	1.05 (0.89–1.23)	0.573	1.22 (1.10–1.36)	<0.001	0.96 (0.84–1.09)	0.493
Prepregnancy BMI <19.8 kg/m^2^	1.12 (1.02–1.22)	0.013	1.19 (1.09–1.31)	<0.001	1.22 (1.10–1.36)	<0.001	1.34 (1.20–1.50)	<0.001
Prepregnancy BMI >24.2 kg/m^2^	1.12 (0.96–1.31)	0.167	1.12 (0.98–1.36)	0.087	1.32 (1.14–1.53)	<0.001	1.36 (1.16–1.58)	<0.001
Conception assisted by reproductive technology	1.29 (0.85–1.96)	0.232	1.17 (0.76–1.80)	0.470	1.57 (0.80–3.59)	0.193	1.39 (0.69–2.79)	0.352
Genetic amniocentesis	1.29 (1.15–1.43)	<0.001	1.24 (1.09–1.41)	0.001	1.49 (1.35–1.65)	<0.001	1.51 (1.33–1.72)	<0.001
Cigarette smoking	0.76 (0.34–1.70)	0.502	0.88 (0.38–2.01)	0.762	2.47 (0.45–13.51)	0.296	2.37 (0.41–13.63)	0.335
Overt diabetes mellitus	0.20 (0.06–0.67)	0.009	0.16 (0.05–0.55)	0.004	1.03 (0.31–3.38)	0.961	0.83 (0.24–2.88)	0.765
Gestational diabetes mellitus	1.09 (0.88–1.36)	0.434	1.16 (0.92–1.46)	0.218	1.40 (1.10–1.78)	0.007	1.31 (1.01–1.69)	0.039
Chronic hypertension	0.58 (0.27–1.26)	0.169	0.66 (0.29–1.48)	0.311	1.24 (0.31–4.95)	0.765	1.09 (0.26–4.65)	0.904
Preeclampsia	0.75 (0.54–1.06)	0.102	0.78 (0.55–1.11)	0.169	0.98 (0.54–1.79)	0.943	0.86 (0.46–1.61)	0.629
Hypothyroidism	0.80 (0.27–2.35)	0.689	0.73 (0.24–2.18)	0.569	1.65 (0.37–7.37)	0.513	1.55 (0.34–7.10)	0.574
Hyperthyroidism	1.67 (0.73–3.81)	0.226	1.72 (0.74–4.02)	0.208	2.17 (0.63–7.40)	0.218	1.65 (0.47–5.80)	0.433
Group B streptococcal colonization	1.23 (1.08–1.40)	0.002	1.23 (1.07–1.40)	0.003	1.30 (1.14–1.48)	<0.001	1.28 (1.12–1.46)	<0.001
Premature rupture of membranes	1.09 (0.75–1.61)	0.646	0.97 (0.65–1.43)	0.860	1.50 (0.82–2.76)	0.193	1.38 (0.73–2.59)	0.322
Oligohydramnios	0.62 (0.38–1.01)	0.055	0.62 (0.37–1.03)	0.065	0.35 (0.07–1.70)	0.194	0.27 (0.05–1.36)	0.112
Polyhydramnios	0.80 (0.27–2.35)	0.689	0.85 (0.28–2.62)	0.783	0.41 (0.04–3.96)	0.442	0.34 (0.03–3.49)	0.366
Birth weight <2500 g	0.55 (0.43–0.71)	<0.001	0.68 (0.51–0.90)	0.008	0.86 (0.60–1.23)	0.401	1.03 (0.68–1.58)	0.879
Birth weight >4000 g	1.05 (0.75–1.48)	0.771	1.05 (0.69–1.58)	0.830	0.92 (0.67–1.25)	0.573	0.77 (0.53–1.12)	0.173
Small-for-gestational age	0.76 (0.67–0.86)	<0.001	0.86 (0.75–1.00)	0.052	0.82 (0.68–0.99)	0.037	0.81 (0.65–1.01)	0.059
Large-for-gestational age	1.03 (0.85–1.25)	0.790	0.91 (0.72–1.16)	0.440	1.09 (0.92–1.30)	0.305	1.07 (0.87–1.31)	0.542
Male fetus	1.07 (0.98–1.16)	0.150	1.06 (0.97–1.15)	0.224	1.02 (0.93–1.12)	0.689	1.01 (0.91–1.12)	0.864
Induction of labor	1.71 (1.55–1.89)	<0.001	2.49 (2.19–2.83)	<0.001	2.31 (2.03–2.64)	<0.001	2.98 (2.55–3.49)	<0.001
Augmentation of labor	0.98 (0.90–1.07)	0.660	1.66 (1.49–1.86)	<0.001	0.96 (0.87–1.05)	0.352	1.46 (1.30–1.64)	<0.001

BMI, body mass index; OR, odds ratio; CI, confidence interval.

^a^ Adjusted for maternal age, prepregnancy body mass index, conception method, genetic amniocentesis, cigarette smoking, overt or gestational diabetes mellitus, chronic hypertension, preeclampsia, hypothyroidism, hyperthyroidism, group B streptococcal colonization, oligohydramnios, polyhydramnios, birth weight <2500 g, birth weight >4000 g, small-for-gestational age, large-for-gestational age, fetal sex, induction and augmentation of labor.

To further clarify the associations between intrapartum epidural analgesia and subsequent operative vaginal and Caesarean deliveries, a multivariable logistic regression analysis was performed to control for the confounding effects of maternal age, prepregnancy BMI, genetic amniocentesis, overt or gestational diabetes mellitus, GBS colonization, augmentation and induction of labor (Table 5). In the nulliparous women, epidural analgesia was a significant risk factor for operative vaginal delivery (adjusted OR 2.14, 95% CI 1.80–2.54), but it was a protective factor against Caesarean delivery (adjusted OR 0.62, 95% CI 0.55–0.69). In the multiparous women, epidural analgesia remained a significant risk factor for operative vaginal delivery (adjusted OR 2.17, 95% CI 1.58–2.97) but not for Caesarean delivery (adjusted OR 1.09, 95% CI 0.77–1.55).

**Table 5:** Association between intrapartum use of epidural analgesia and different modes of delivery.

Delivery mode	Nulliparous women: epidural vs. no epidural analgesia				Multiparous women: epidural vs. no epidural analgesia			
	Unadjusted OR (95% CI)	*P*	Adjusted OR^a^ (95% CI)	*P*	Unadjusted OR (95% CI)	*P*	Adjusted OR^a^ (95% CI)	*P*
Spontaneous vaginal delivery	0.94 (0.86–1.04)	0.227	1.06 (0.96–1.17)	0.232	0.56 (0.45–0.70)	<0.001	0.65 (0.52–0.82)	<0.001
Operative vaginal delivery	2.23 (1.89–2.64)	<0.001	2.14 (1.80–2.54)	<0.001	2.37 (1.75–3.23)	<0.001	2.17 (1.58–2.97)	<0.001
Caesarean delivery	0.73 (0.66–0.81)	<0.001	0.62 (0.55–0.69)	<0.001	1.24 (0.89–1.72)	0.196	1.09 (0.77–1.55)	0.639

OR, odds ratio; CI, confidence interval.

^a^ Adjusted for maternal age, prepregnancy body mass index, genetic amniocentesis, overt or gestational diabetes mellitus, group B streptococcal colonization, augmentation, and induction of labor.

## Discussion

An ideal method to study the effects of epidural analgesia on the modes of delivery and perinatal outcomes is to conduct a randomized, controlled trial. However, because the use of epidural analgesia is rapidly increasing, it will be increasingly difficult to recruit participants to random allocation between epidural analgesia and no epidural analgesia or non-pharmacological analgesia. Other difficulties in conducting a randomized, clinical trial include the enormous expense, non-adherence to protocols, absence of comparable analgesic methods, and impossibility of blinding during the performance of the trial and the assessment of the results. These limitations underlie the strength and importance of studies such as ours, which used a cohort design with control of confounding variables.

In this study, a low prepregnancy BMI, a history of genetic amniocentesis, GBS colonization, augmentation and induction of labor were independent factors for the use of epidural analgesia in both the nulliparous and multiparous women. A high prepregnancy BMI and gestational diabetes mellitus were additional independent factors for epidural analgesia in the multiparous women. It is not clear why women with these characteristics were more likely to request epidural analgesia during their trials of labor. However, maternal age appeared to play a role because the frequency of genetic amniocentesis, gestational diabetes mellitus, a high prepregnancy BMI, and GBS colonization increased with maternal age [18]. Moreover, several of these factors have been associated with an increased risk of Caesarean delivery or adverse perinatal outcomes. Therefore, it is important to consider the confounding effects of these factors when evaluating the associations between intrapartum epidural analgesia and different modes of delivery and adverse perinatal outcomes.

In accordance with most previous studies [2–5,9–11,6,7], we demonstrated that epidural analgesia was associated with an increased rate of operative vaginal delivery and a longer labor duration in the first and second stages of labor in both the nulliparous and multiparous women. In contrast, the relationship between epidural analgesia and Caesarean delivery was more complex; epidural analgesia was associated with a lower rate of Caesarean delivery in the nulliparous women, while no such association was noted in the multiparous women. These results were different from several previous reports that claimed epidural analgesia increased the risk for Caesarean delivery [9,10,7]. It is possible that we controlled for the confounding effects of maternal demographic and pregnancy characteristics; thus, the association between epidural analgesia and Caesarean delivery could be evaluated more objectively. Alternatively, approximately 70% of nulliparous women and 45% of multiparous women in our hospital used epidural analgesia during their trials of labor. It is likely that a high rate of intrapartum epidural analgesia was associated with active management of labor, which would neutralize any negative effects caused by epidural analgesia. Indeed, more than 50% of our parturients underwent augmentation of labor, reflecting a policy of active management of labor in our obstetric care.

In addition to the Caesarean delivery rate, lower frequencies of non-reassuring fetal heart rate and severe preeclampsia as the indications for Caesarean delivery were identified in the nulliparous women who were administered epidural analgesia compared with the women who were not administered epidural analgesia. The explanations for these associations are not clear. Pain and cognitive activity in the early stages of labor have been noted to be important contributors to labor efficiency and obstetric outcomes. It has been reported that women with higher levels of pain or distress-related thoughts were more likely to undergo a Caesarean delivery compared with women with less pain or thoughts conducive to coping [19]. Moreover, women with distress-related thoughts had five times the incidence of abnormal fetal heart rate patterns in active labor than women with thoughts of coping. It is possible that epidural analgesia reduced the levels of pain and anxiety, with a more profound effect on nulliparous women, thus decreasing the risk of abnormal fetal heart rate tracing and Caesarean delivery.

Epidural analgesia also has an effect on maternal blood pressure [6]; women with preeclampsia who were administered epidural analgesia had lower blood pressure compared with the women who were not administered epidural analgesia [20]. Nevertheless, it is doubtful that epidural analgesia changes the rate of severe preeclampsia, because the syndrome likely has its origins long before labor. Instead, it is possible that epidural analgesia may impact this one sign of preeclampsia and delay diagnosis or that women with severe preeclampsia had contraindications to epidural analgesia, such as thrombocytopenia.

In this study, we determined that the women who were administered epidural analgesia during trials of labor had similar rates of adverse perinatal outcomes compared with the women who were not administered epidural analgesia, with the exception that an increased rate of 1-minute Apgar scores less than 7 was noted in the nulliparous women who were administered epidural analgesia. This difference was most likely related to an increased rate of operative vaginal delivery by vacuum extraction.

This study had several strengths compared with previous reports. One was the large sample size and high frequency of intrapartum epidural analgesia in our population. Therefore, the differential effects of epidural analgesia on the modes of delivery and adverse perinatal outcomes between nulliparous and multiparous women could be studied. Another strength of this study lay in its ability to adjust for as many confounding factors as possible, as well as its use of patient interviews and medical record data, rather than birth certificate data; thus, the factors associated with intrapartum use of epidural analgesia and the relationships between epidural analgesia and different modes of delivery and adverse perinatal outcomes could be objectively investigated.

In contrast, several limitations of our study merit attention. First, the study had the attendant limitations of a hospital-based study. The techniques of epidural analgesia, management of labor, and criteria for surgical intervention can differ between hospitals. As a result, the applicability of our results to different clinical settings should be carefully evaluated. Second, this study was limited by its observational and retrospective design. Information regarding the cervical dilatation when epidural analgesia was administered, number of top-ups, prior experience, and complications of epidural analgesia was lacking. Nevertheless, there is mounting evidence that demonstrates there is no increased risk of Caesarean delivery or instrumental vaginal delivery for women who receive early epidural analgesia at cervical dilatations of 3 cm or less compared with late epidural analgesia [21]. Third, the present study lacked information regarding the psychological and socio-economic characteristics of the study population. Previous studies have shown that epidural analgesia was used more often in women with private insurance, higher educational levels, higher incomes, and severe subjective pain [14,22,23]. How these factors might affect the relationships between epidural analgesia and the modes of delivery remain unclear. Finally, there are no non-Asians in this study; thus, the comparison of our findings with other ethnicities is limited.

Another limitation of this study is that it covers a 13-year period; therefore, it may be argued that some obstetric practice and treatment policies that changed during the course of the study may have contributed to the differential changes in outcomes. However, this scenario is unlikely because of the following reasons. First, with the exception of the implementation of the new criteria for the diagnosis of gestational diabetes mellitus recommended by the International Association of Diabetes and Pregnancy Study Groups and a protocol of antenatal magnesium sulfate therapy for neuroprotection in preterm delivery <32 weeks of gestation in 2011, there were no additional changes in obstetric care during the time period that would be expected to confound the study results. Second, we excluded deliveries before 37 weeks of gestation and controlled for the confounding effect of gestational diabetes mellitus when we assessed the association between epidural analgesia and different modes of delivery. Third, the clinical structure of the department did not significantly change.

## Conclusions

Our study has two new and important findings, which we suggest have clinical implications. First, we demonstrated that intrapartum epidural analgesia has differential effects on the modes of delivery between nulliparous and multiparous women and that it is not associated with adverse perinatal outcomes. Second, we demonstrated that epidural analgesia was not associated with an increased risk for Caesarean delivery in the multiparous women and was a protective factor against Caesarean delivery in the nulliparous women, despite the prolongation of the labor duration and increased risk for operative vaginal delivery. These findings are particularly important for nulliparous women because they are more anxious regarding labor and delivery compared with multiparous women, have no prior experience with epidural analgesia, and are the main population that requests epidural analgesia. Obstetric care providers would benefit from this information to enhance their counseling regarding the intrapartum use of epidural analgesia for nulliparous women.
